# Definition and evolution of right ventricular dysfunction in critically ill COVID-19 patients

**DOI:** 10.1186/s13613-022-01055-z

**Published:** 2022-08-30

**Authors:** Minesh Chotalia, Mansoor N. Bangash, Jaimin M. Patel, Dhruv Parekh

**Affiliations:** 1 Birmingham Acute Care Research Group, University of Birmingham, Birmingham, UK; 2 Department of Anaesthetics and Critical Care, Queen Elizabeth Hospital, Birmingham, UK

**Keywords:** RV dysfunction, Echocardiography, COVID-19, Acute myocardial injury

We read with great interest the single-centre study conducted by Jansson et al. [1] in 74 ICU patients with COVID-19, that detailed the evolution of acute myocardial injury [AMI; defined by high sensitivity troponin (HS-Tn) rise] across the first 2 weeks of ICU stay and its association with left and right ventricular dysfunction (RVD), diagnosed from a transthoracic echocardiogram (TTE) performed within 72 h of ICU admission.

Prospective TTE studies in consecutive COVID-19 patients are sparse due to the time and expertise required to perform serial examinations during a period of immense clinical burden. The authors should be commended for undertaking this impressive feat. However, their conclusion that RVD did not correlate with mortality or with AMI warrants further consideration.

The timing of TTE (within 72 h) preceded the peak incidence of AMI (denoted by peak HS-Tn levels) which occurred 1 week following ICU admission. It is, therefore, unsurprising that RVD did not associate with AMI, as the timing of the TTE was unlikely to be temporally related to AMI development. If the TTE was performed at the time of myocardial injury, then an association may well have been observed. For example, in a study of 172 ICU patients with COVID-19 who had TTE’s performed later on during ICU stay (median 7 days) in patients with a HS-Tn rise, RVD was associated with significantly higher HS-Tn levels [2].

An interesting observation by Jansson et al. was that the peak of NT-ProBNP (median 4 days) preceded the peak of HS-Tn (median 7 days). Indeed, we hypothesised that progressive RV dilation, although initially compensatory via the Frank–Starling mechanism, when severe, may eventually precipitate subendocardial ischaemia, RV systolic impairment and reduced RV forward flow [2, 3]. This is supported by the finding of RV dilation *with* systolic impairment being the RV phenotype most strongly associated with mortality and having the highest HS-Tn levels [2, 3]. Nonetheless, in a large multicentre retrospective study [4] where TTE was performed at a median of 3 days, RVD defined as acute cor pulmonale (RV dilation and septal dyskinesia) was also associated with mortality.

The lack of association between RVD and mortality/AMI in this study may, therefore, reflect how RVD was defined: as a composite of any 2 variables denoting either RV dilation (RV:LV end diastolic area (RV:LVEDA) > 0.6) or RV systolic impairment (RV fractional area change (FAC) < 35%, tricuspid annular plane systolic excursion < 17 mm, (TAPSE), free wall strain > − 20%, S prime < 6 cm/s). This definition is not validated, not recommended in any guidelines and has not been used in previous studies. Inspecting Table 2, patients defined as having ‘RVD’ by Jansson et al., had no difference in RV size (RV:LVEDA) or RV FAC compared to the non-RVD group. Without any difference in RV size, whether this cohort reflects ‘true’ RVD or not remains to be seen. For example, we found that RV systolic impairment alone was not associated with mortality or high HS-Tn levels. Only when it was combined with RV *dilation* was the association with mortality and HS-Tn observed. Other RVD definitions that have associated with mortality in COVID-19 ICU patients also employ metrics of RV size [4, 5]. These include acute cor pulmonale [4] (as defined above) and RV dilation with evidence of venous congestion [5] (CVP > 8; ESICM definition) and neither of these definitions were utilised in this study.

Clearly, selection bias may also play a role in the discrepant findings between this and other studies [2–5]. Jansson et al.’s study included all consecutive patients with COVID-19 admitted to ICU (albeit 20 patients were missed due to clinical burden), whereas most other studies have included cohorts where TTE was performed in selected patients [2–5]. However, as evident in their study, patients without AMI had a 0% incidence of RVD and a 0% 30 day mortality rate. Therefore, AMI diagnosis may be an appropriate way of stratifying which patients should receive TTE examination. Indeed, in our cohort, patients with COVID-19 ARDS that did not receive a TTE also had low HS-Tn levels and low mortality [3]. It was, therefore, unlikely that these patients had RVD and not performing a TTE in this cohort was unlikely to affect the association between RVD and mortality or myocardial injury.

The final reason that RVD may not associate with mortality or AMI in this study is that the study is underpowered to detect this association, with only *n*=13 patients diagnosed with RVD.

## Conclusions

Ultimately, the association of RVD with myocardial injury and mortality in critically ill COVID-19 patients is difficult to elucidate without large, prospective studies performing *serial* TTE examinations to describe the evolution of RVD across ICU stay. These studies must also employ validated definitions of RVD. Until this is done, it would be reticent to conclude that RVD does not associate with myocardial injury or mortality in COVID-19 or other critically ill cohorts, especially given the breadth of other literature suggesting that it does.

## Data Availability

Not applicable.

## Declarations

### Completing interests

On behalf of all authors, there is no completing interest.
